# Comparison of Antero-Posterior Lip Position in Most Favored Facial Profile of Jaipur Population

**DOI:** 10.7759/cureus.27774

**Published:** 2022-08-08

**Authors:** Kuldeep Sharma, Mridula Trehan, Shikha Singh, Heeral Mahlawat, Priyanka Kenkare, Arya Jayavarma S

**Affiliations:** 1 Dentistry: Orthodontics and Dentofacial Orthopaedics, Rajasthan Dental College & Hospital, Jaipur, IND; 2 Dentistry: Orthodontics and Dentofacial Orthopaedics, NIMS Dental College & Hospital, Jaipur, IND; 3 Dentistry: Orthodontics and Dentofacial Orthopaedics, Braces & Smiles, Mumbai, IND; 4 Dentistry: Orthodontist, Indira Gandhi Institute of Dental Sciences, Puducherry, IND

**Keywords:** jaipur, orthodontics, retrusion of lip, protrusion, nemotech, anteroposterior lip positions

## Abstract

Background

The enhancement of facial esthetics is one of the essential goals of orthodontic treatment. The concept of an excellent facial profile can differ between two individuals of the same population. Esthetics is a very vital component in Orthodontic diagnosis and treatment planning. Orthodontic treatment aims to achieve facial harmony by stabilizing the occlusion and pleasing facial and dental esthetics. Therefore, the characteristics of a pleasing, well-balanced face and a functioning occlusion should be evaluated.

Aim

This study aimed to determine preferable Antero-posterior lip positions in the Jaipur population and determine the range of anteroposterior lip positions as evaluated by orthodontists, patients, and professionals from a series of different lip positions in facial silhouettes, and compare their assessments.

Materials and Methods

The sample comprised 50 subjects (25 males & 25 females) from Jaipur. All angular and linear measurements were recorded for both males and females separately. The mean value of those readings was placed in NEMOTECH cephalometric software, and an average profile construction was done. This average profile will determine the lip fullness/ facial profile of males and females of the Jaipur (Rajasthan) population. A series of 7 profile silhouettes for males and seven profile silhouettes for females was developed by altering the lip positions (protrusion & retrusion in 2mm increments till 6 mm from the average profile) parallel to Frankfort horizontal plane (F-H) plane. Constructed facial silhouettes (seven male and female) were evaluated by (50) Orthodontists, (50) oral surgeons, general dentists (50), and non-specialists population (100). Evaluators were asked for the best profile in each gender from 1 to 7 in order of their preference.

Result

On calculating the standard deviation values, the profile selected as most favored for females is 2.33 by orthodontists, on the contrary, the standard deviation for Oral Surgeons, General Dentists & Non-specialists is 2.04, 2.14, and 2.12 respectively. It was found that profile retruded -2mm for males was the most favored, and profile retruded -4mm for males was second most favored. Average Profile /the profile without retrusion or protrusion was selected by orthodontists as the most favored profile. Profile retruded -2mm was the most favored profile for males clinically significant. It was found that profile retruded -2mm for females was the most favored, and profile retruded -4mm for females was second most favored. Profile retruded -2mm was the most favored profile for females by Orthodontists, General Dentists & non-specialists. Profile retruded -4mm was the most favored for females by Oral Surgeons. overall made insignificant.

Conclusion

The most favored facial profile is -2mm (profile 1) retruded lip posture in both males and females. According to Orthodontists most favored facial profile is 0mm (profile 6) average profile in males and -2mm (profile 1) retruded lip position in females. According to non-specialists most favored facial profile is -2mm (profile 1) retruded lip position in both males and females.

## Introduction

Modern society attributes a lot to esthetics, particularly facial esthetics [1]. Perception of facial beauty may differ among different populations and change with time. Most people seek orthodontic treatment to enhance their facial appearance [2]. Despite this, present-day concepts of what constitutes good facial appearances are controversial and have not been clearly formulated. Esthetics, described as the science of beauty in nature, is a vital part of orthodontic diagnosis and treatment planning [3]. Every person desires a pleasant face; a pleasant face is directly related to the relationship of various soft tissue structures. The relation of these soft tissue structures is again related to the underlying osseous structures and muscles. Therefore, the dental, skeletal, and neuromuscular systems should be in harmony to obtain a balanced, soft tissue relationship. Hence, it will be essential to recognize and estimate the parameters of sensing and maintain a stable face and balanced occlusion [4]. This can be a task challenge due to the characteristics of a relative face changing and are influenced by age, gender, ethnicity, culture, and personality [5].

The main objective in the treatment phase of orthodontics is to attain facial esthetic harmony by stabilizing the dentition and pleasing facial and dental esthetics. Facial esthetics is perceived differently by professionals and non-specialists [5]. Due to these differences, the Orthodontist may have difficulty assessing the patient profile and treatment plan acceptable to the patient. This is more obvious when the treatment is likely to modify the soft tissue profile [6].

The Orthodontist does and must have a prominent role in shaping the esthetic destiny of a patient's profile. There is an increasing tendency today for the doctor, general dentist, Orthodontist, or plastic surgeon to dominate the esthetic considerations of his particular treatment completely. The patient and his family are seldom asked to express their esthetic viewpoint or concept. Overlooking the patient's expectation of treatment could result in patient discontent, despite good outcomes from the orthodontic treatment [2]. Orthodontists have primarily reviewed horizontal lip position as one of the factors that play a role in determining facial beauty [7].

The motto of the study is to compare the anteroposterior lip position of preferred profiles evaluated in the soft-tissue range by Orthodontists, General Dentists, Dental Students, and non-specialists from a series of varying lip positions using facial silhouettes. In addition, observation of the facial view contents considered acceptable for both gender will be done by Orthodontists, Oral surgeons, General Dentists, and Non-specialists.

This study addresses the following questions to check the most favored profile and lip position and how the facial profile concept varies among Orthodontists, Oral surgeons, General dentists, and Non-specialists

## Materials and methods

**Figure 1:**
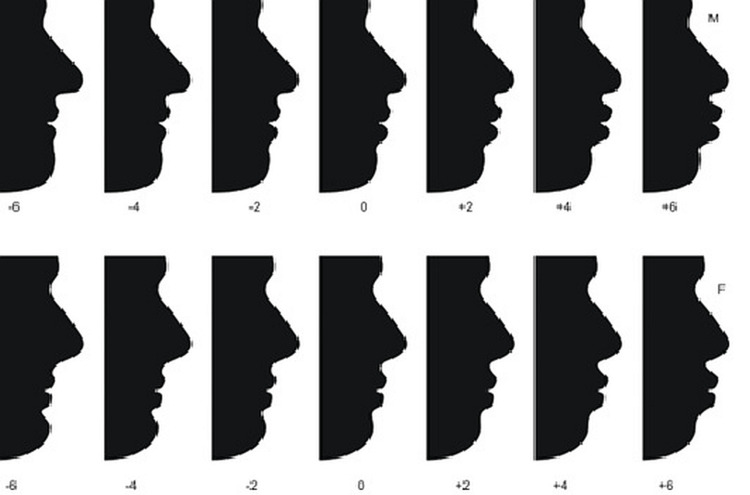
Silhoutes of the profiles The facial silhouettes were randomly placed presenting varied lip fullness measurements.

The sample consisted of 50 subjects (25 males & 25 females) from Jaipur with ideal occlusion having been accepted from the review board of Rajasthan dental college having review number RUHS/RDCH/DISSERTATION/BATCH-2011-14/2325. Readings of all angular and linear measurements were recorded for both males and females separately of the age group of 20 -30 yrs, and the mean value of those readings was placed in NemoTec cephalometric software (Parque Científico Leganés Tecnológico, SPAIN), and an average profile construction was done. This average profile determined the lip fullness/ facial profile of males and females of the Jaipur (Rajasthan) population. A series of 7 profile silhouettes for males and females were developed by altering the lip change in positions like protrusion & retrusion in 2mm increments till 6 mm from the average profile) parallel to the F-H plane. Vertical references SN-7 below sella nasion line with pogonium line minimum 1.5mm of upper lip were not taken in this study. Linear measurements were made with a millimeter ruler from the most anterior point of the lip and the most anterior point of the lower lip with measurements made with Nasolabial and labiomental angle adapting over lip morphology sella nasion line with pogonium line are recorded. Vertical references were not taken in this study, as shown in Figure 1.

Evaluation of facial profile

Constructed facial profiles (7 male and 7 female) were evaluated by Orthodontists (50), Oral surgeons (50), General dentists (50), and Non-specialists (100), non-specialists are patients participating in the study who were ideally educated about the study. They were using parameters for this study and readings calculated and measured for the study. Scoring of best profile: Scorers were asked to score for the best profile in each collection of profiles from 1 to 7 in order of their necessity.

Pieces of information are reviewed to evaluate the importance of the following individualistic components as mostly favored by both gender profiles, whether the assessor was an Orthodontist, Oral surgeon, General dentist, or non-specialist. Levels of the most significant selected profile choice of the Orthodontist, oral surgeon, general dentist, and non-specialists and between gender profiles with the help of the Chi-Square test( Pearson).

Data was retrieved to evaluate the importance of the following individualistic components as mostly favored by both gender profiles, whether the assessor was an Orthodontist, Oral surgeon, General dentist, or non-specialist. Levels of the most significant selected profile choice of the Orthodontist, oral surgeon, general dentist, and non-specialists and between gender profiles with the help of Chi-Square test( Pearson) and to find actual significant value by Kruskal Wallis test.

## Results

**Figure 2:**
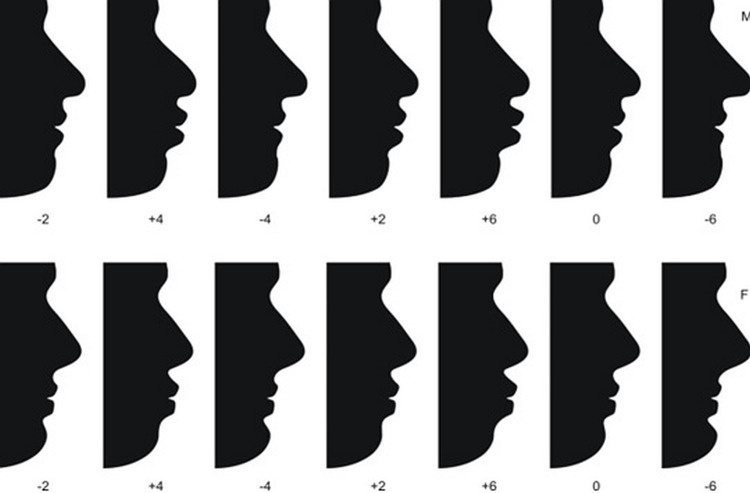
Randomly arranged protruded and retruded lip position (silhouettes) made from the software used.

The results of this study are based on the frequency (mode) as depicted by the percentage of a particular profile as the most preferred profile in each series selected by Orthodontists, Oral surgeons, General dentists, and non-specialists. These results are explained in tables 1-3 and Figure 2.

**Table 1:** Mean values of selected parameters for average profile construction. E-Line: esthetic line

Selected parameters	E-Line		Sn–Pog		Nasolabial angle	Z-angle	
	UL	LL	UL	LL		UL	LL
Male total values	-75.1	-11.1	114.6	109.6	2532.3	1845.1	1767.2
Mean value	-3.01	-0.45	4.19	4.37	101.29	73.80	70.69
Female total values	119.98	29.8	101.9	91.7	2510.1	1874.3	1799.4
Mean value	4.79	1.19	4.07	3.67	100.40	74.97	71.78

**Table 2:** Sequentially arranged profile values

S.NO.	MEASUREMENTS	DECREASED VALUES	ORIGINAL VALUES	INCREASED VALUES	S.NO.	MEASUREMENTS	DECREASED VALUES	ORIGINAL VALUES
SERIES1	LIP SIZE (mm)	-6	-4	-2	0	+2	+4	+6
SERIES 2	LIP SIZE (mm)	-6	-4	-2	0	+2	+4	+6

**Table 3:** Randomly arranged profile values

S.NO.	MEASUREMENTS	PROFILE 1	PROFILE 2	PROFILE 3	PROFILE 4	PROFILE 5	PROFILE 6	PROFILE 7
SERIES 1 MALE	LIP SIZE (MM)	-2	+4	-4	+2	+6	0	-6
SERIES 2 FEMALE	LIP SIZE (MM)	-2	+4	-4	+2	+6	0	-6

It was found that profile 1 for males is most favored, profile 3 for males is second most favored, profile six is most favored among orthodontists, and profile 1 is most favored among oral surgeons, general dentists & non-specialists (Table 4).

**Table 4:** Male Favorable Profile selected by all groups

Occupation	Facial Profile							
	1	2	3	4	5	6	7	Total
Orthodontist	17	0	8	2	0	23	0	50
Oral Surgeon	16	0	15	1	0	13	5	50
Gen. Dentist	20	0	13	10	0	5	2	50
Non-specialists	18	0	17	9	1	5	0	50
Over all	71	0	53	22	1	46	7	200

It was found that profile 1 in females was the most favored, profile 3 for females was the second most favored, profile 1, for females was most favored by Orthodontists, General Dentists & Non-specialists and profile 3 for females was the most favored by Oral Surgeons (Table 5).

**Table 5:** Female favorable profile selected by all groups

Occupation	Facial Profile							
	1	2	3	4	5	6	7	Total
Orthodontist	22	0	6	3	3	16	0	50
Oral Surgeon	14	0	18	4	0	13	1	50
Gen. Dentist	28	3	11	4	0	4	0	50
Non-specialists	22	0	17	9	0	2	0	50
Over all	86	3	52	20	3	35	1	200

It was found that profile retruded -2mm for males was the most favored, and profile retruded -4mm for males was second most favored. Orthodontists selected the average Profile /the profile without retrusion or protrusion as the most favored profile where the E-line reference line was used to differentiate protruded and retruded lips. Profile retruded -2mm was the most favored profile for males by Oral Surgeons, General Dentists & non-specialists. Table 6 shows the significance levels of the most preferred profile among the selected group among various male profiles. Pearson Chi-square test was used to calculate significance levels. No statistically significant difference (P > .05) was reported in all series of profiles selection.

**Table 6:** Male favorable profile on retrusion & protrusion

Occupation	Facial Profile							
	-6	-4	-2	0	+2	+4	+6	P value
Orthodontist	0	8	17	23	2	0	0	0.00
Oral Surgeon	5	15	16	13	1	0	0	0.00
Gen. Dentist	2	13	20	5	10	0	0	0.00
Non-specialists	0	17	18	5	9	0	1	0.00
Overall	7	53	71	46	22	0	1	0.00

It was found that profile retruded -2mm for females was the most favored, and profile retruded -4mm for females was second most favored. Profile retruded -2mm was the most favored profile for females by Orthodontists, General Dentists & Non-specialists. Profile retruded -4mm was the most favored for females by Oral Surgeons. Table 7 shows the significance levels of the most preferred profile among the selected group among various female profiles. Pearson Chi-square test was used to calculate significance levels. No statistically significant difference (P > .05) was reported in all series of profiles selection.

**Table 7:** Female Favorable Profile on retrusion & protrusion

Occupation	Facial Profile							
	-6	-4	-2	0	+2	+4	+6	Total
Orthodontist	0	6	22	16	3	0	3	50
Oral Surgeon	1	18	14	13	4	0	0	50
Gen. Dentist	0	11	28	4	4	3	0	50
Non-specialists	0	17	22	2	9	0	0	50
Overall	1	52	86	35	20	3	3	200

Table 8 shows the mean, standard deviation & median value difference of various male profiles selected by orthodontists, Oral Surgeons, General Dentists & Non-specialists. Table 8 shows no significant difference in mean values of Orthodontists, General Dentists & Non-specialists as values are -1.24,-1.68,-1.60, respectively. In contrast, a marked difference was reported in the mean value of Oral Surgeon, which is -2.40. On calculating the standard deviation values Profile selected as most favored for males is 1.61 by orthodontists. On the contrary standard deviation for Oral Surgeons, General Dentists & Non-specialists is 2.02,2.30,2.42. The median value For orthodontists is -1, indicating that the most favored profile for orthodontists is -1 mm retrusion of the lip. Median Value For Oral Surgeons, General Dentist, and Non-specialists are -2, indicating that their most favored profile is -2 mm lip retrusion. The probability distribution value for various profiles for males is 0.059, indicating no significant difference in the selection of profiles by various groups.

**Table 8:** Favorable male mean, median, and standard deviation values

Occupation	N	Mean	Standard Deviation	Median	Kruskal Wallis Test	
					H value	p value
Orthodontist	50	-1.24	1.61	-1	8.009	0.059
Oral Surgeon	50	-2.40	2.02	-2		
Gen. Dentist	50	-1.68	2.30	-2		
Non-specialists	50	-1.60	2.42	-2		

Table 9 shows the mean, standard deviation & median value difference of various female profiles selected by orthodontists, Oral Surgeons, Gen. Dentists & Non-specialists. The table shows no significant difference in mean values of Oral surgeons, General Dentists & Non-specialists as values are -1.96,-1.60,-1.88, respectively. At the same time, a marked difference was reported in the mean value of Orthodontists, which is -0.88. On calculating the standard deviation values Profile selected as most favored for females is 2.33 by orthodontists. On the contrary standard deviation for Oral Surgeons, General Dentists & Non-specialists is 2.04, 2.14, and 2.12. The median Value For Orthodontists, Oral Surgeons, General dentists, and Non-specialists is -2, indicating that the most favored profile for them is -2 mm retrusion of the lip. The probability distribution value for various profiles for males is 0.059, indicating no significant difference in the selection of profiles by various groups.

**Table 9:** Favorable female mean, median, and standard deviation values

Occupation	N	Mean	Std. Deviation	Median	Kruskal Wallis Test	
					H value	p value
Orthodontist	50	-0.88	2.33	-2	8.202	0.054
Oral Surgeon	50	-1.96	2.04	-2		
Gen. Dentist	50	-1.60	2.14	-2		
Non-specialists	50	-1.88	2.12	-2		

## Discussion

Current conception in treatment and diagnosis aims at the facial esthetics and alignment of the various facial characters. Malocclusion, stable tooth position, and facial principles are influenced by the total content of muscle as well as bone, position, and soft tissue structures. General functions, morphology, and lip posture are mainly concerned by orthodontists [7]. A vital part has always been a soft tissue profile assessment in diagnosis and treatment planning. The selection of treatment is the position of lips remarkably determined. Changing the lip position can also be an Orthodontic treatment. Hence, one of the most vital soft tissue lip positions has become ideal, as it influences the occlusion, stable tooth position, and appearance of the face [7]. Significant degrees of variation in the perception of beauty among persons, racial groups, countries, and according to socioeconomic status. The Orthodontist must locate the normal facial profile from the abnormal. Deal on Asian countries of lip profiles in studies proved that displaced inward upper and lower lips were the norm for esthetic Japanese females, as shown by Nakahara et al. [8]. Another study by Loi et al. [9] revealed that the Japanese raters preferred more displaced inward lip positions, so the facial convexity decreased than the protruded lip position as the facial convexity increased. Displaced lip position was also preferred for Asian populations like Singapore and China [8,10]. Nose and chin characteristics vary with each race [11]. Ricketts developed his principles for Caucasians, whereas Sushner applied his principles for the Black population. Thus, during treatment planning, the application of principles to the soft tissue of one population would be different for another. 

This has usually been studied in full-face and profile views of Facial profiles and features. Powell and Rayson [12] used the three-fourth facial profile in addition to a more complex interpretation of the face. Since the facial esthetic complexities could not be totally expressed in a single analysis method, a facial profile view provides necessary details for diagnosis and treatment planning of dentofacial problems. Facial profiles have been mostly evaluated using cephalometric or photometric references, linear, proportional, and angular measurements or combinations. Various studies [13-19] on facial attractiveness in orthodontic journals and books have concentrated on the profile outline by using tracings or casts instead of profile photographs.

The motto of this study was to analyze the role of the lips in achieving a balanced facial profile and perception of varied facial profiles by different selected groups and to determine their importance based on sex. For this cast, outline shape profiles were constructed based on the originally selected profile. Using silhouette profiles to analyze facial profile views may be considered an alternative to other means of facial calculations. Silhouette in the study simplified the profile representation, focused mainly on the facial profile outcome, and avoided bias by eliminating the influence of variable factors such as skin tones, hair model, layer-up, and facial functions. This study utilized cast outline or "mask type" profiles instead of pictorial representations of the living persons whose skin complexion, hairstyle, and expression can be obvious, avoiding bias. Differences between the profile preferences for both genders were observed during the statistical analysis. Other studies involved mostly females or less number of male subjects that it was unable to obtain sex differences in facial profile preferences. 

This study established that in lip position estimation, the preference for lip protrusion and retrusion was not equal Profile 1 was preferred as the most favored profile in males and females when all four evaluator groups were counted. However, individual evaluators' judgment varies in the selection of profiles for both males and females.

Orthodontists preferred an average profile of 6 (0mm) for males with standard lip size but good lip fullness, but for females, Orthodontist selected profile 1 (-2mm) retruded lip position than normal. Oral surgeons preferred profile 1 (-2mm) for males with a decrease in lip size, but for females, the Oral surgeon selected profile 3 (-4mm) for retruded lip position than usual. General dentists preferred profile 1 (-2mm) for both males and females, retruded lip position than usual. Non-specialists preferred profile 1 (-2mm) for both males and females, retruded lip position than normal. The concept of facial esthetics between Orthodontists, Oral surgeons, general dentists, and non-specialists varied due to the knowledge and involvement in the current field also differed in each group, as stated by Secord and Backman [20], because of similarity in the situation where their opinion different from Wylie's 20 learnings gains importance, which stated that in the matter of facial profile view, Orthodontic specialist never holds their judgment superior over Oral surgeons, general dentists, and non-specialists. They concluded that non-specialists preference for the human profile was as good as the Orthodontist's and often better, as it was not modified by orthodontic means. Therefore, it is vital in orthodontic and orthognathic treatment planning to include and emphasize the patient's desires and expectations. Overlooking a patient's desire without adequate communication may result in discontent, even though the treatment results are effective and good.

The design of this study's nose, lips, and chin positions were such that changes in size might be valued in choices. It is very important for the Orthodontist who treats malocclusions and alters facial profile to observe the favored variations as the lips, nose, and chin relationship exits harmoniously. Patients with more protrusive teeth, prominent chin and nose, and total lip thickness are acceptable for a balanced facial profile. Out of such patients, if there is a dilemma regarding the extraction or non-extraction treatment, then a non-extraction treatment plan should be preferred. However, in case of severe arch length discrepancy, where extraction is the only choice, the treatment plan with extractions can be carried out. The harmony of facial features and esthetics should be the most vital objective in any treatment mechanics. Solid adherence to skeletal norms or occlusion alone and planning treatment to these norms may not be the best extent of achieving good results.

In the past literature, differences in facial profiles among males and females were largely avoided, mostly since it was not validated by statistical analysis or demonstrated by a scientific study. Orthodontic treatment plans were mostly confined to the movement of teeth in the alveolar bone. Therefore the change in the soft-tissue profile and range of esthetic enhancement was limited by the area of the teeth surrounding the bone. New treatment procedures like orthopedic forces and orthognathic surgery are now available, which help change the position of teeth, supporting structures, and facial profile.

The role and inter-relation of soft and hard-tissue variation with orthognathic have been verified. This helps the Orthodontist accurately predict the change in the profile that can be done by surgical intervention. Orthognathic surgery, along with orthopedic force treatment, has proved clinically important.

Orthodontist treatment capabilities currently include - conventional orthodontic treatment, orthopedic treatment, and orthognathic surgery. Currently, the orthodontic field has been updated with adequate diagnostic tools and treatment possibilities so that the patients can get the best desirable facial esthetics and harmonious functional occlusion. Facial profile development of soft tissues is the outcome of various changes in the structures of the face and interaction between the hard and soft tissue. Hence, it is vital to observe the difference in the growth of facial soft tissue with more attention paid to the soft tissue areas. It has to be well established that the profile of the face and shapes change with orthodontic and orthognathic treatment. Therefore, while developing a comprehensive treatment plan, facial changes required should be figured out well in advance. These changes must be evaluated to determine whether or not they are desirable. Thus, there is a need to study dentofacial relations and their effect on esthetic balance and facial contours. However, many studies define a beautiful face and its various facial components, and the definition changes about the change in ethnic groups or countries. The study of facial esthetics and balanced soft tissue profile remains important since facial profile preference varies among ethnic groups.

Each individual has unique facial features and differs from others, so they cannot get similar treatment results among patients regarding facial esthetics. Therefore, treatment in orthodontics should concentrate more on improving the present esthetic characteristics and enhancing facial profile, which is altered due to dentoskeletal mal relationship.

The study limitations are the considerably small sample size, and the particular population study cannot give us a generalized statement.

## Conclusions

Improving facial esthetics is the main aim of orthodontic treatment. Recently, significant focus has been on facial esthetics by both patients and orthodontists. Traditional orthodontic treatment planning gave more importance to functional occlusion and its stability, thereby overlooking the patient's desire for enhanced facial appearance. In the study, Silhouette simplified the profile representation by assessing facial esthetics and eliminating other factors influence. There was a clear difference in preference of some components of facial profiles of both genders by the Orthodontists and patients, while some facial features were not. Some features which are more discernable to one may not look good to the other gender differences. Variations in facial preference were noticed in both genders; along with Orthodontists, Oral surgeons, General dentists, or non-specialists, the most favored facial profile is -2mm (profile 1) displaced inward lip position in both genders. According to Orthodontists most favored facial profile is 0mm (profile 6) average profile in males and -2mm (profile 1) retruded lip position in females. According to the Oral surgeon most favored facial profile is -2mm (profile 1) retruded lip position in males and -4mm (profile 3) retruded lip position in females. According to the General dentist, the most favored facial profile is -2mm (profile 1) displaced inward lip position in both genders. According to non-specialists most favored facial profile is -2mm (profile 1) displaced inward lip position in both genders.
